# A prodrug strategy for sustained release of lactic acid from silicone elastomer vaginal rings

**DOI:** 10.1016/j.ijpharm.2025.126120

**Published:** 2025-08-26

**Authors:** Yahya H. Dallal Bashi, Xinyu Zhao, Clare F. McCoy, Narender Kumar, Natalia Teleshova, Dolores J. Lamb, José A. Fernández Romero, Abigail Meyer, Patrick Barnable, Meropi Aravantinou, Osaretin E. Asowata, Melissa A. White, Alexander J. Travis, Lisa B. Haddad, Xin Shen, Vicky-Leigh Young, Peter Boyd, R. Karl Malcolm

**Affiliations:** a College of Pharmacy, University of Sharjah, Sharjah, United Arab Emirates; b School of Pharmacy, Queen’s University Belfast, Belfast, BT9 7BL, UK; c Center for Biomedical Research, Population Council, New York, NY 10065, USA; d Department of Department of Surgery/Pediatric Urology Div., Children’s Mercy Research Institute, University of Missouri-Kansas City School of Medicine, Kansas City, MO 64108, USA; e Science Department, Borough of Manhattan Community College, The City University of New York, New York, NY 10007, USA; f Baker Institute for Animal Health, Department of Public & Ecosystem Health, Cornell University, USA

**Keywords:** Lactide, Hydrolysis, Non-hormonal contraception, Multipurpose prevention technology (MPT), Human immunodeficiency virus (HIV), Herpes simplex virus (HSV), Lactobacillus, Intravaginal ring, Bacterial vaginosis

## Abstract

Lactic acid is the most abundant organic weak acid in the healthy human vagina and plays a pivotal role in maintaining an acidic vaginal environment protective against exogenous bacteria and viruses. However, in dysbiotic or non-optimal vaginal environments, significantly decreased concentrations of lactobacilli result in reduced lactic acid production, increased vaginal pH, and enhanced risk of sexually transmitted infections (including human immunodeficiency virus), and bacterial vaginosis. Various gel-based products are marketed to administer lactic acid vaginally for the treatment of bacterial vaginosis and non-hormonal contraception, and there is interest in developing vaginal ring products for sustained/controlled release of lactic acid. However, lactic acid is not compatible with the most common addition-cure type of silicone elastomer used to manufacture vaginal rings; the carboxylic acid group inhibits the hydrosilylation reaction used to cure the elastomer system. Here, we report that DL-lactide—a racemic mixture of (R,R)-D-lactide and (S,S)-L-lactide, in which the dilactide molecules are cyclic lactones derived from esterification of two molecules of lactic acid—can be successfully incorporated into and released from addition-cure medical grade silicone elastomer vaginal rings. Following release of lactide from the rings into an aqueous medium, the lactide molecule rapidly hydrolyses to produce only lactic acid. We demonstrate that lactic acid (i) is formed f release of lactide from the rings; (ii) inhibits sperm motility, (iii) inhibits replication of HIV-1 and HSV-2, and (iv) is active against *Gardnerella vaginalis* (one of the causative organisms responsible for bacterial vaginosis) but not lactobacillus (associated with optimal human vaginal health). The results support the inclusion of lactide as a lactic acid prodrug in next-generation multi-purpose contraceptive silicone elastomer vaginal rings.

## 1. Introduction

**Fig. 1.**
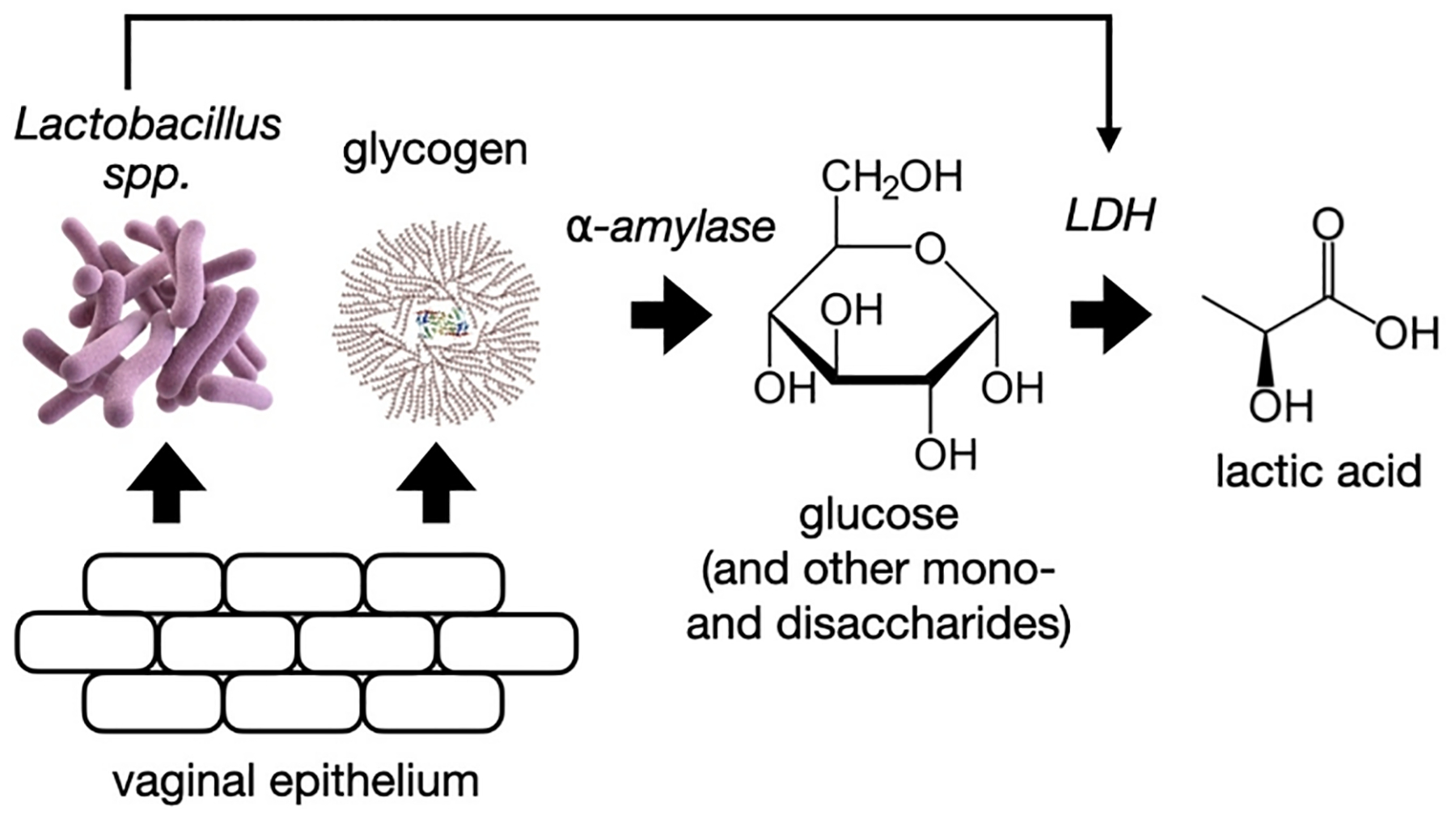
The production of lactic acid in the human vagina.

The healthy human vaginal microbiota is unique among mammals—including primates most genetically related to us—in that it is dominated by communities of certain species of Lactobacillus bacteria (*L. iners, L. crispatus, L. gasseri, or L. jensenii*) (Fuochi et al., 2017; Ravel et al., 2011). Typically, more than 70 % of the resident bacteria in the healthy human vagina are lactobacilli, compared to < 1 % in other mammals (Miller et al., 2016). In the presence of estrogen, vaginal epithelial cells produce glycogen, which is subsequently hydrolysed to form mono- and disaccharides—including glucose—by the action of local α-amylases derived from both bacterial and human origins (Fig. 1) (Nunn et al., 2020). The endogenous lactobacilli in the healthy vagina then metabolize glucose to lactic acid (Fig. 1). Consequently, lactic acid is the most abundant organic weak acid in both the healthy (55–111 mM; 4.9–10 mg/mL) and the dysbiotic (11–20 mM; 1–1.8 mg/mL) human vagina (Aldunate et al., 2013; O’Hanlon et al., 2011; Schwecht et al., 2023) and plays a pivotal role in maintaining an acidic (pH 4–5) vaginal environment protective against pathogenic bacteria and viruses (Boskey et al., 2001; Miller et al., 2016; Tachedjian et al., 2017). For example, in its uncharged protonated form, lactic acid (i) has potent and irreversible activity against human immunodeficiency virus type 1 (HIV-1) (Aldunate et al., 2013; Hearps et al., 2017; Tyssen et al., 2018), (ii) acidifies the vagina following dissociation, which is useful for the treatment of bacterial vaginosis (Anstey Watkins et al., 2019; Mendling et al., 2022; O’Hanlon et al., 2011; Plummer et al., 2021a), (iii) enhances cervicovaginal epithelial barrier integrity by promoting tight junction protein expression (Delgado-Diaz et al., 2022), and (iv) shows spermicidal activity (Su and Vincent, 2022).

Various aqueous vaginal gel products containing lactic acid are currently marketed (Table 1), primarily for regulating vaginal pH to help treat bacterial vaginosis, but also for on-demand contraception since sperm are inactivated at acidic pH (although, after intercourse, the buffering activity of the seminal fluid temporarily abolishes the normal acidic pH for a few hours allowing the sperm to maintain functionality and allowing fertilization to occur) (Masters and Johnson, 1966; Tevi-Bénissan et al., 1997).

Lactic acid dosing with these gel products ranges from 90–225 mg per application. Compared to these single-dose gel products, longer-term maintenance of an acidic vaginal pH might be better achieved by use of a vaginal ring device releasing lactic acid, which would obviate the need to administer relatively large gel quantities. Verstraelen *et al*. reported a thermoplastic matrix-type vaginal ring fabricated from ethylene vinyl acetate and methacrylic acid-methyl methacrylate copolymers and loaded with 150 mg DL-lactic acid (Verstraelen et al., 2016). This ring released ~ 37 mg lactic acid total over seven days, based on residual content analysis following safety evaluation in healthy nulliparous premenopausal volunteers (Cunningham et al., 1994; Wain, 1998). Other vaginal ring devices fabricated via hot-melt extrusion from thermoplastic polyurethanes and containing up to 30 % w/w DL-lactic acid have also been reported (Verstraete et al., 2017).

**Fig. 2.**
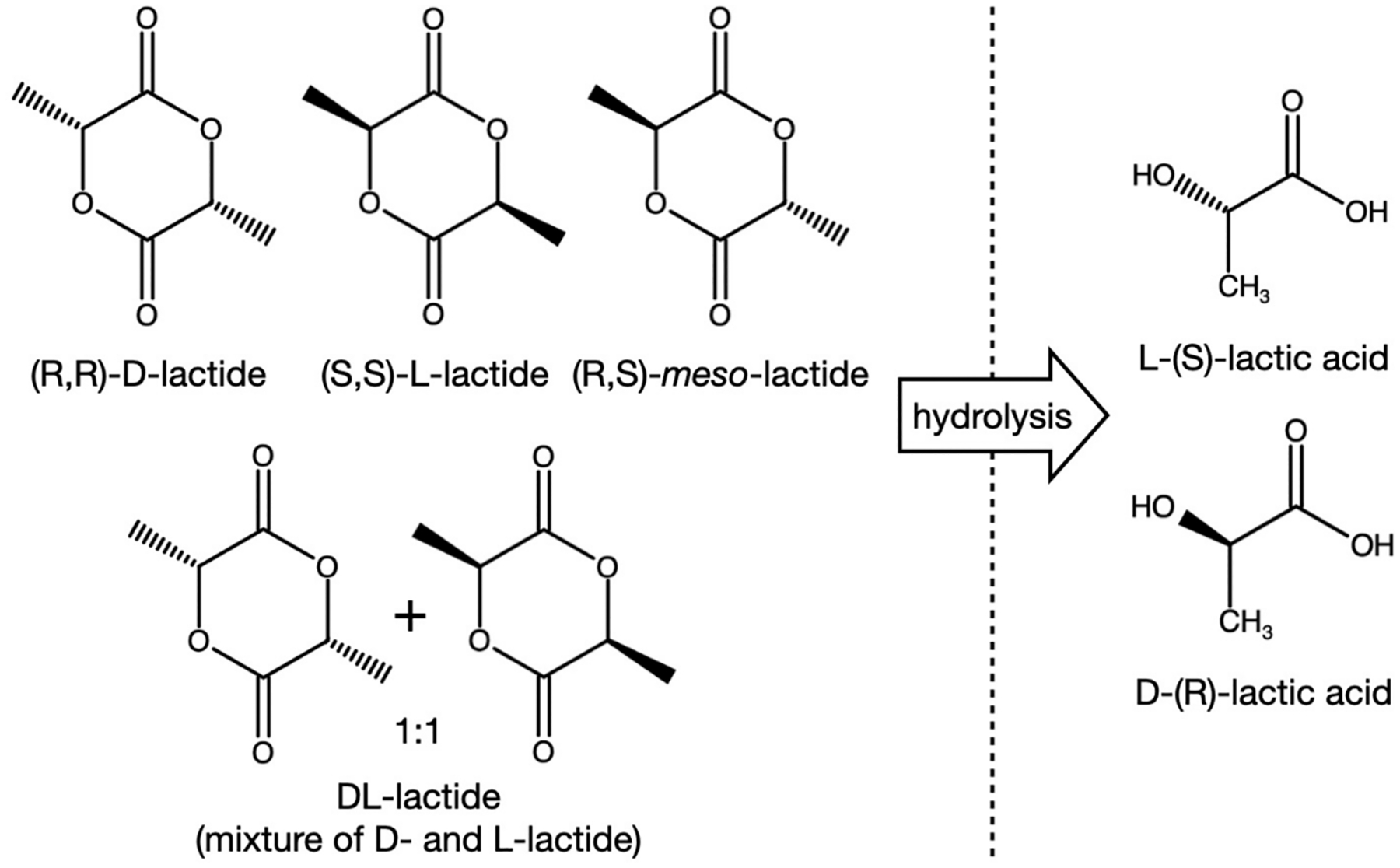
Chemical structures and different stereochemical forms of lactic acid and lactide.

In this manuscript, we show that lactic acid is incompatible with addition cure silicone elastomers, the most common type of silicone elastomer used to manufacture rings. As with many molecules containing carboxylic acid functional groups, the lactic acid inhibits the platinum-catalysed hydrosilylation crosslinking reaction (Malcolm et al., 2016). As part of efforts to develop a lactic acid releasing silicone elastomer vaginal ring and to increase the quantity and duration of lactic acid release beyond those reported previously for thermoplastic rings (Verstraelen et al., 2016; Verstraete et al., 2017), we assessed the potential of using DL-lactide (3,6-dimethyl-1,4-dioxane-2,5-dione)—a racemic mixture of (R,R)-D-lactide and (S,S)-L-lactide, where the lactide molecules are cyclic lactones derived from esterification of two molecules of lactic acid (Fig. 2) such that the carboxylic acid groups are masked—as a lactic acid prodrug. Specifically, we assessed DL-lactide compatibility with addition cure silicone elastomers (via oscillatory rheology, thermal analysis, and ring manufacture) and assessed *in vitro* release of lactide from rings and its hydrolysis to form lactic acid. This lactic acid prodrug strategy may be particularly useful in developing new silicone elastomer vaginal ring products for improved treatment of bacterial vaginosis and multipurpose prevention technology (MPT) products in which the lactide component is combined with other actives to additionally prevent unintended pregnancy, HIV infection, and other sexually transmitted infections (Boyd et al., 2016; Fetherston et al., 2013; Malcolm and Fetherston, 2013; Shen et al., 2025; Smith et al., 2017; Zhao et al., 2023b, 2023a). A similar prodrug strategy was previously leveraged in a drug-releasing vaginal ring; 17β-estradiol-3-acetate—an ester prodrug of 17β-estradiol, the principal circulating estrogen in the human female—is the drug substance in the Femring^®^ product (Woolfson et al., 1999). To further bolster the lactide concept, we also evaluated the activity of lactic acid/DL-lactide against sperm motility, human HIV-1, herpes simplex virus type 2 (HSV-2), *Gardnerella vaginalis* (one of the causative organisms responsible for bacterial vaginosis), and *Lactobacillus* species widely associated with human vaginal health.

## 2. Materials and Methods

### 2.1. Materials

Liquid silicone rubber (Silbione^®^ LSR-4350, product code A-225–50, Lot number 51009032) was supplied by Factor II, Inc. (Lakeside, CA, USA). DDU-4320 silicone elastomer was supplied by NuSil Technology LLC (Carpinteria, CA, USA). DL-lactide (purity: 99 %; Lot number: 10225071) and lactic acid (crystalline powder, purity: 98 %; Lot number: 10237498) were purchased from Alfa Aesar (Lancashire, UK). Potassium dihydrogen orthophosphate was purchased from VWR International Ltd. (Dublin, Ireland). HPLC-grade acetonitrile, and phosphoric acid 85 % w/w in water were purchased from Merck Life Scientific Ltd. (UK). A Millipore Direct-Q 3 UV Ultrapure Water System (Watford, UK) was used to obtain HPLC-grade water. Milled (micronized) DL-lactide powder was produced using a Pulverisette 2 Mortar Grinder (contact pressure = 0–10 daN; Fritsch^®^, Idar-Oberstein, Germany) for 7 min and then sieved to < 100 μm (Retsch^®^, Germany). DSC and TGA pans and lids (standard aluminium and Tzero) were purchased from TA Instruments (Wilmslow, UK). Herpes simplex virus 2 strain G (ATCC, Manassas, VA, USA) and Vero cells (ATCC) were used in the HSV-2 plaque assay (Ashley, 1995). Vero cells were grown in complete medium Dulbecco Modified Eagle medium (DMEM; ThermoFisher Scientific, Waltham, MA) supplemented with 10 % heat-inactivated fetal bovine serum (FBS; ThermoFisher Scientific, UK) and with 50 U/mL penicillin and 50 μg/mL streptomycin (ThermoFisher Scientific, UK). HIV-1 BaL (ABI, Eldersburg, MD, USA) and the HeLa cell-derived TZMbl reporter cell line (AIDS Reagent Program) was used in multinuclear activation of an indicator (MAGI) assay (Begay et al., 2011). TZMbl cells were grown in DMEM supplemented with 10 % heat inactivated FBS and with 100 U/mL penicillin and 100 μg/mL streptomycin. *Lactobacillus jensenii* (*L. jensenii*) strain 62G (ATCC) and *L. crispatus* strain VPI 7635 (ATCC) were used in anti-*Lactobacillus* assays. Lactobacilli were cultured in Man, Rogosa and Sharpe (MRS) broth (Becton Dickinson, Franklin Lakes, NJ, USA) and colonies were grown anaerobically on Lactobacillus Selection (LBS) agar. *Gardnerella vaginalis* strain 594 (ATCC) was used in the anti-*G. vaginalis* assay. *G. vaginalis* was cultured in Brucella Broth supplemented with 10 % horse serum (BBSH) (Sigma Aldrich, St. Louis, MO). Colonies were grown on Columbia CNA Agar (Negoen, Lansing, MI, USA) supplemented with 1 g/L yeast extract (MP Biomedicals, Irvine, CA), 3 g/L beef extract (MP Biomedicals), 4 mg/L amphotericin B (Sigma Aldrich, UK), and 5 % whole human blood (New York Blood Center, New York, NY, USA). For cellular, viral and bacterial tests, L-(+) lactic acid—one of two possible isomeric forms of lactic acid (Fig. 2), and produced by vaginal epithelial cells, lactobacilli, and other bacteria (Beghini et al., 2015)—was purchased from Spectrum Chemical (New Brunswick, USA).

### 2.2. Microscopy

Digital microscopy was performed on samples of un-milled (as supplied) and milled DL-lactide using a Keyence VHX-700F Digital Microscope (Keyence Ltd., UK) fitted with a RZ 20–200x wide-range zoom lens. Each material was dusted onto the surface of adhesive tape to provide a thin layer of powder for particle size and shape analysis.

### 2.3. Particle size distribution

The particle size distribution (PSD) of milled DL-lactide (<100 μm) was measured by laser diffraction using a Mastersizer 3000 (Malvern, UK) fitted with an AERO S dispersion accessory. ~150 mg of material was added to the Venturi disperser. The air pressure, feed rate and hopper gap were set to 3 Bar(g), 45 % and 2.5 mm, respectively. Five measurements were performed achieving an RSD < 3 % for Dv50 and an RSD < 5 % for Dv10 and Dv90 (International Organization for Standardization, 2020).

### 2.4. Thermogravimetric analysis (TGA)

The thermal properties of milled DL-lactide powders and silicone elastomer samples containing milled DL-lactide were analysed using a Thermal Advantage Model TGA Q50 (TA instruments, New Castle, PA, USA). Samples (10 g) of blank uncured silicone elastomer LSR-4350 and DL-lactide-loaded (10 % w/w) uncured silicone elastomer LSR-4350 were prepared by speedmixing 1:1 ratio of parts A and B (with specified quantities of drug powders) using a dual asymmetric centrifuge mixer (SpeedMixer^™^ DAC 150 FVZ-K, Hauschild, Germany) at 3000 rpm for 10 s. For DL-lactide powder samples (1–3 mg), TGA analyses were conducted using a standard ramp heating from 20 to 180 °C in both an open pan and a pan with a pinhole lid. A custom TGA method comprising ramp heating to 115 °C, isothermal at 115 °C for 3 min, followed by ramp heating to 180 °C at 10 °C/min was applied to the blank and drug-loaded uncured silicone elastomer LSR-4350 samples (3–5 mg) to better mimic ring injection molding conditions. Weight percentages were plotted as a function of temperature. All TGA analyses were conducted in triplicate.

### 2.5. Differential scanning calorimetry (DSC) analysis

DSC testing was conducted using a DSC25 instrument (TA instrument, Wilmslow, UK) under a nitrogen atmosphere (50 mL/min). Powders of milled DL-lactide (1–3 mg) were weighed accurately in sealed Tzero pans. Standard ramp heat experiments from 20 to 150 °C at 10 °C/min were conducted. To further investigate the influence of the heating protocol used during ring manufacture, a custom heating protocol to mimic changes in temperature during vaginal ring manufacture was used: (i) rapid heating to 115 °C and equilibration at 115 °C; (ii) isothermal at 115 °C for 5 min (longer than the 3 min injection molding); (iii) rapid cooling to 20 °C and equilibration at 20 °C; and (iv) heating from 20 to 150 °C at 10 °C/min. Blank LSR-4350 silicone elastomer ring samples and rings loaded with 20 or 30 % w/w milled DL-lactide were cut, weighed (typically 5–10 mg) and placed into sealed Tzero pans and heated from 20 to 150 °C at 10 °C/min. All samples were tested against an empty reference pan. All thermal transitions (mainly crystalline melting) were recorded as onset values determined from the heat flow signal. Values were reported as a mean ± standard deviation of three measurements.

### 2.6. Oscillatory rheology

Rheological studies were performed to assess how DL-lactide and lactic acid affected the curing characteristics of an addition-cure silicone elastomer system (DDU-4320). Silicone elastomer mixes (3.0 g) were prepared by accurately weighing Part A and Part B (1:1) silicone elastomer (plus the required amount of DL-lactide or lactic acid, as required) into sealed polypropylene Speedmixer^™^ containers. Silicone mixes were hand-mixed for 30 s followed by mixing at 3000 rpm for 30 s in a DAC-150 FVZ-K SpeedMixer^™^. Oscillatory rheology was performed using an AR2000 rotational rheometer (TA Instruments, UK). Immediately after mixing, the silicone mixes were placed on the rheometer’s lower stationary plate using a plastic spatula, and the upper plate (40 mm crosshatch) lowered to produce a 1000 μm gap between the plates. Excess silicone was removed from the plate assembly prior to initiation of the experiment. The time taken for sample placement and initiation of the experiment was approximately 30 s. An oscillatory frequency of 1 Hz and an amplitude of 1 % strain (8.7 Pa) were used for all analyses (McConville et al., 2010). Isothermal cure was performed on drug-free (blank), DL-lactide-loaded (1, 2.5, 5, and 11 % w/w) and lactic acid-loaded (1 % w/w) DDU-4320 silicone elastomer samples at 40, 60 and 80 °C. (These lactide loadings do not match the loadings described later for ring devices, as the studies were conducted at very different times by different researchers. The catalyst in addition cure silicone elastomers is very sensitive to inhibition by certain extraneous substances. In our experience, if cure inhibition is not observed at loadings < 10 %, it is highly unlikely that cure inhibition will be observed at higher loadings.) Values for storage modulus (G′), loss modulus (G″) and tan delta (tanδ) were measured as a function of time. All rheology experiments were performed for 80 min to allow sufficient time for complete curing at each cure temperature.

### 2.7. Ring manufacture

Matrix-type LSR-4350 vaginal rings were manufactured using custom ring molds (outer diameter 57.6 mm, cross sectional diameter 7.8 mm) fitted to a laboratory-scale injection molding machine. Prior to injection molding, the temperature of the closed mold assembly was equilibrated at 115 °C for at least 30 min. For the placebo (blank) ring formulation, LSR4–350 Parts A and B (1:1) were weighed accurately in a 100 g polypropylene container and speedmixed (3000 rpm, 30 s). The blank active mix was transferred into SEMCO cartridges fitted with metal nozzles, ready for injection molding. For the DL-lactide-loaded ring formulations, LSR-4350 Part A and Part B premixes (75 g) were prepared separately by accurately weighing appropriate quantities of LSR-4350 into a Speedmixer^™^ container followed by mixing (3000 rpm, 5 s, DAC-150 FVZ-K Speedmixer^™^) to settle the silicone on the bottom of the container. Lactide was then added, hand-mixed, and then speedmixed (3000 rpm, 30 s). Immediately prior to injection molding, the Part A and B premixes were sequentially added to a large plastic Speedmixer^™^ container followed by hand mixing, speedmixing (350 rpm, 30 s), and a final speedmixing step (1800 rpm, 60 s). The active mix was transferred to SEMCO cartridges fitted with metal nozzles. The cartridge was then inserted into a caulking gun (Makita^®^ DCG 180, UK), and the material injected into the mold at speed 4 until excess material was seen at the vent points of the ring mold. All ring formulations were cured at 115 °C for 3 min. Three lactide-loaded LSR-4350 ring formulations (10 %, 20 % and 30 % w/w; L10, L20 and L30, respectively) were manufactured and tested in this study.

### 2.8. Ring weight and geometry

Ring weight, colour, outer diameter (OD) and cross-sectional diameter (CSD) were recorded both before and after *in vitro* release testing. Values for OD and CSD were measured using digital callipers (Mitutoyo, Hampshire, UK); care was taken to ensure that the rings were not compressed or distorted during measurement. Following *in vitro* release testing, measurements were recorded for both D30 and D30 dried rings. Wet D30 rings were removed from the *in vitro* release media and blotted dry with laboratory tissues before measurement. D30 dried rings were dried at 50 °C in an Incu-Shake FL16–2 incubator (SciQuip, Shropshire, UK) for up to 21 days until no further reduction in weight was observed (recorded weight within 0.02 g of the previous measurement). Dried rings were returned to ambient temperature (21 °C) before measurements were recorded.

### 2.9. Durometer hardness testing

Durometer hardness was measured using a Checkline RX-DD-M Type M digital durometer (Shore M scale) held in an RX-OS-3 test stand (Checkline Europe, Birmingham, UK). A custom ring holder, fabricated to match the outer diameter of the ring geometry being tested, was placed onto the base plate of the RX-OS-3 stand. The ring samples (n = 3 rings per formulation) were placed into the appropriate ring holder and four individual measurements recorded at different locations on the ring surface.

### 2.10. Ring compression testing

Compression testing of rings was performed using an EZ-LX Universal Tester (Shimadzu, UK) fitted with a custom-fabricated compression jig. A lower plate with multiple grooves into which the rings loosely fit was mounted on the lower fixed platform of the tester. An upper plate with identical grooves was mounted on the upper arm of the tester (such that the centrelines of lower and upper grooves were aligned). A single ring was placed in the central groove of the holder and the upper plate adjusted downward (according to the outer diameter of the ring) until the ring was held in a vertical position. The crosshead was further lowered so that the ring was held in 4 mm pre-compression prior to the test commencing. Rings were compressed by 5, 10, 15 and 20 mm using a test speed of 5 mm/s and the force (N) required for compression at each distance recorded.

### 2.11. In vitro release testing

*In vitro* release testing for DL-lactide-loaded rings was assessed over 30 days (n = 3 per formulation). On day 0 (D0), rings were individually placed into 100 mL plastic, screw-cap flasks containing 20 mL of ultrapure water (pH 5.5, to represent bacterial vaginosis) and stored in a Incu-Shake FL16–2 orbital shaking incubator (37 °C, 60 rpm, 25 mm orbital throw; SciQuip, Shrewsbury, UK). The 20 mL release medium in each flask was sampled and completely replaced daily except for weekends when 60 mL of media was added to maintain sink conditions. Release samples were analysed for both DL-lactide and lactic acid concentrations using reverse phase HPLC with UV detection.

### 2.12. Ring content assay

Content assay measuring DL-lactide and lactic acid was performed only for the 30 % w/w DL-lactide LSR-4350 rings (n = 4), primarily to assess the accuracy/precision around lactide loading and its stability in the formulation. Rings were weighed and cut into small pieces. The ring pieces were transferred into individually labelled 1 L glass Duran^®^ bottles containing 1000 mL of ultrapure water. Bottles were sealed and placed in an Incu-Shake FL16–2 orbital shaking incubator (50 °C, 80 rpm, 25 mm orbital throw). After 21 days, the bottles were removed from the incubator and allowed to cool to room temperature for at least 1 h. After cooling, a 1–2 mL aliquot from each flask was transferred to HPLC vials and analysed against standard solutions of known DL-lactide and lactic acid concentrations using HPLC with UV detection.

### 2.13. HPLC analysis

*In vitro* release and content samples were analysed for DL-lactide and lactic acid using a Waters Alliance HPLC system (Waters e2695 Separations Module and Waters 2489 dual λ detector; Waters Limited, Ireland). Samples (50 μL) were injected onto a Thermo Scientific BDS Hypersil^™^ C18 column (150 × 4.46 mm, 3 μm particle size) maintained at 35 °C and fitted with a guard column. Isocratic elution was performed at 1.3 mL/min using a mobile phase of 4 % v/v acetonitrile and 96 % v/v potassium phosphate buffer (7.7 mM; pH 3.0) with a run time of 5 min. Lactic acid and DL-lactide were detected at a wavelength of 210 nm after 1.8 and 3.7 min, respectively.

### 2.14. Anti-sperm activity of lactide /lactic acid

Stock solutions containing lactic acid and DL-lactide were prepared in 5 % w/v DMSO in saline 12–24 hr before testing for anti-sperm activity. With informed consent, Weill Cornell Medicine institutional review board approval, and institutional oversight, semen samples were collected from paid donors with an abstinence period of 2–7 days as recommended by WHO 6 (World Health Organization, 2021). Inclusion criteria for participation only required that they follow the required protocol for semen collection by masturbation as described in WHO (World Health Organization, 2021). Fresh human sperm samples were obtained early in the morning with onsite collection on the day of the experiment and allowed to liquify for 30 min with the experiment beginning between 30 min and no longer than an hour after collection. Immediately prior to the experiment, lactide/lactic acid solutions of various concentration were prepared via serial dilution with 5 % DMSO in saline. For each dilution, 50 μL fresh semen sample were mixed with 200 μL solution or 5 % DMSO saline control and allowed to sit for 5 min. Two cover-slipped slides each of 10 μL of sample were then manually counted under a microscope (2 × 100 total sperm; 400 sperm per API) by two different laboratorians trained in performing a routine semen analysis and movement of each sperm classified as either motile with forward progression, twitching, vibrating, moving with no progression or totally non-motile based on their type of flagellar movement or lack thereof. Each experiment was repeated on different days using different donors to account for natural variations in semen/sperm properties. The percent motility for each experimental condition was calculated by normalizing the fraction of motile sperm in the experimental groups to the fraction of motile sperm in the saline control treated group, setting 100 % to the average fraction of motile sperm from control experiments. A dose–response curve using percent motility versus lactic acid concentration was utilized to estimate the half-maximal effective concentration (EC_50_) values using GraphPad Prism (version 10, GraphPad, Boston, MA). Similar experiments were performed at Cornell University (Ithaca, NY), with Cornell institutional review board approval and institutional oversight, to investigate whether observed effects of lactic acid on sperm motility were due to changes in pH, or some other effect.

### 2.15. Anti-HSV-2 assay

HSV-2 was incubated with different concentrations of lactic acid (0.03–2.10 mM) or only the complete medium (control). For this purpose, the virus at 10^4^ plaque forming units (PFU/mL) was mixed with an equal volume of each lactic acid concentration diluted in complete medium. The samples were incubated for 30 min at 37 °C, after which the virus was titered using the HSV-2 plaque assay (Ashley, 1995). Each lactic acid concentration and control were tested in triplicate in two independent experiments. A dose–response curve using percent of viral replication versus lactic acid concentration was used to estimate the EC_50_ values using GraphPad Prism.

### 2.16. Anti-HIV-1BaL assay

HIV-1BaL was incubated (30 min, 37 °C) with equal volumes of different concentrations of lactic acid in saline (0.13–17.20 mM) or saline alone (control). After incubation, samples were diluted 1/10 in complete medium before TZMbl cells were challenged with ~ 0.001–0.002 multiplicity of infection (MOI) HIV-1BaL. The virus was titered as previously described (Begay et al., 2011). Following 72 h incubation, TZMbl cells were stained with 5-bromo-4-chloro-3-indolyl-β-D-galactosidase yielding an insoluble blue product and fixed. The positive cells were quantified using ImmunoSpot^®^ analyser (Cellular Technology Limited, Shaker Heights, OH). Each lactic acid concentration and control were tested in triplicate in three independent experiments. A dose–response curve using percent of viral replication versus lactic acid concentration was used to estimate the EC_50_ using GraphPad Prism.

### 2.17. Anti-lactobacillus assay

*L. jensenii* and *L. crispatus* were grown (separately) in suspension in MRS broth for 18–24 h. Then, bacteria at 5 × 10^5^ CFU/mL were incubated with different concentrations of lactic acid (13.88–888 mM) in the presence or absence of 10 or 30 % human AB serum (Sigma Aldrich) or MRS broth alone (untreated control) for 30 min at 37 °C (Moncla and Hillier, 2005). This method allows for the detection of rapid killing of the test bacteria. After incubation, 25 μL of each condition was plated in duplicate on LBS agar plates and cultured anaerobically for 2–3 days. LBS agar plates containing penicillin (100 U/mL) and streptomycin (100 μg/mL) were included as a positive control. To confirm the inoculum, untreated controls were diluted 1:100 and plated on LBS agar plates (expected colony count is ~ 125 per plate).

### 2.18. Anti-Gardnerella vaginalis assay

*G. vaginalis* were grown in suspension in Brucella broth supplemented with 10 % horse serum (BBHS, Sigma Aldrich, St. Louis, MO) for 48 h. Then, bacteria at 5 × 10^5^ CFU/mL were incubated with different concentrations of lactic acid (13.88–888 mM) or BBHS alone (untreated control) (Moncla and Hillier, 2005). After incubation, 25 μL of each condition was plated in duplicate on blood agar plates and cultured anaerobically for 48 hr. Blood agar plates containing 32 mg/L metronidazole (Sigma) were included as a positive control. To confirm the inoculum, untreated controls were diluted 1:100 and plated on blood agar (expected colony count is ~ 125 per plate).

### 2.19. Statistical analysis

Statistical analyses were performed using one-way ANOVA, followed by post-hoc analysis using the Tukey-Kramer multiple comparisons test. Statistical significance is defined as p < 0.05. Analysis was conducted using GraphPad Prism (v 10.2.3; GraphPad Software, LLC.).

## 3. Results and discussion

### 3.1. Particle size of lactide

**Fig. 3.**
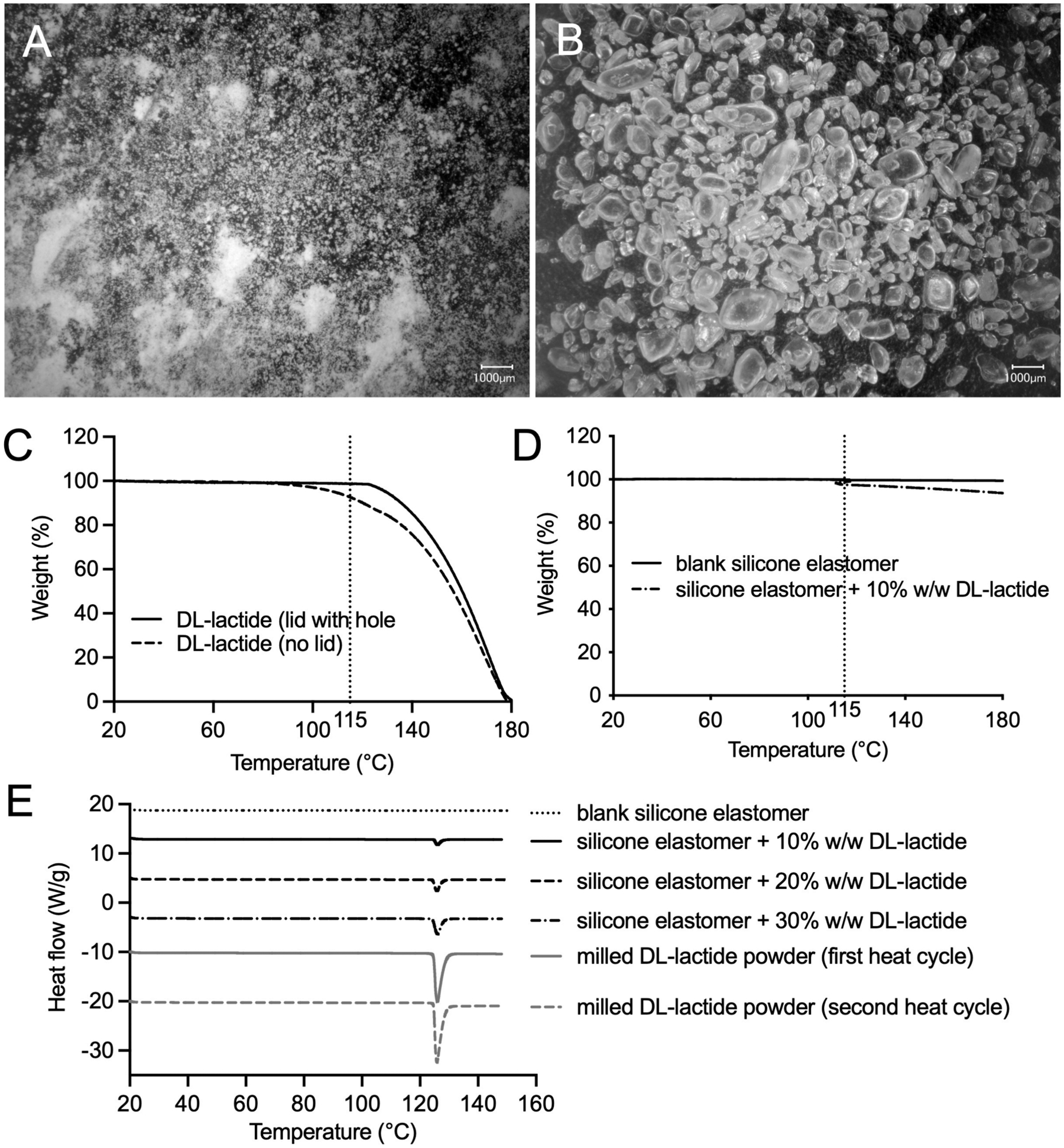
Representative micrographs (×20 magnification with glare removal filter applied) of (A) freshly milled DL-lactide and (B) DL-lactide as supplied (unmilled). TGA thermograms of (C) milled DL-lactide heated from 20 to 180 at 10 °C/min, and (D) uncured blank LSR-4350 silicone elastomer and uncured LSR-4350 silicone elastomer containing 10 % w/w milled DL-lactide following heating to 115 °C, isothermal at 115 °C for 5 min, and finally ramp heating to 180 °C at 10 °C/min (to mimic cure conditions). From a time perspective, the dotted line at ~ 115 °C in panel C represents the transition from heating to isothermal mode. The rings were manufactured at 115 °C. (E) Representative DSC thermograms for DL-lactide and silicone elastomer samples. Blank silicone elastomer showed no thermal transitions; DL-lactide alone showed a large endothermic melting peak in both normal ramp heat mode (heated 20–150 °C at 10 °C/min) and following a custom thermal method (heat to and equilibrate at 115 °C; isothermal at 115 °C for 5 min; cool to and equilibrate at 20 °C; and finally heat 20–150 °C at 10 °C/min). DSC thermograms are also presented for LSR4350 silicone elastomer ring formulations having different loadings of DL-lactide.

Representative micrographs of unmilled and milled DL-lactide are presented in Fig. 3. The unmilled material appeared highly crystalline with large particles (~250–2000 μm) (Fig. 3B). Milled material (Fig. 3A) showed mostly small primary particles and some larger agglomerates. The particle size of the milled material was too small to measure using digital microscopy; by laser diffraction, it measured 2.4 μm (Dv10), 8.3 μm (Dv50), 33.4 μm (Dv90) and 3.740 (span value). Micronized DL-lactide is most suitable for incorporation into silicone elastomer mixes and manufacture of vaginal rings by injection molding, since it ensures drug content uniformity and prevents larger particles blocking injection and vent ports in the injection molds. All marketed vaginal ring products contain micronized drug.

### 3.2. Thermogravimetric analysis

TGA is useful in evaluating the mass loss processes in silicone elastomer vaginal rings (for example, due to drug volatility or instability), particularly at the elevated temperatures used for injection molding (Murphy et al., 2016). TGA traces for milled DL-lactide (20–180 °C, 10 °C/min) are presented in Fig. 3C. In pans with no lids, DL-lactide started to lose weight from ~ 80 °C and weight loss was complete by 180 °C. For pinhole lid samples, the onset temperature for DL-lactide weight loss was 122 °C, close to the reported melting temperature (122–126 °C) (D’Avila Carvalho Erbetta, 2012; Emelyanenko et al., 2009). DL-lactide and lactic acid boil at 140 °C at 8 mm Hg (314 °C at 760 mm Hg) and 267 °C at 760 mm Hg, respectively; thus, lactic acid is more volatile. However, given the nitrogen atmosphere used for TGA analysis, the observed weight loss is due to evaporation of lactide, rather than hydrolysis of lactide to lactic acid and subsequent evaporation of lactic acid (D’Avila Carvalho Erbetta, 2012). Thus, in the absence of the silicone elastomer, loss of lactide via evaporation begins at > 80 °C, significantly below both its melting temperature and the ring manufacturing temperature.

We anticipated that this lactide loss would reduce when mixed with the silicone elastomer. Milled DL-lactide was mixed with the uncured Part A + Part B silicone elastomer mix and analysed by TGA using a custom heating protocol designed to mimic the temperature profile during ring manufacture. Briefly, uncured blank LSR-4350 silicone elastomer samples and 10 % w/w DL-lactide (milled)-loaded uncured LSR-4350 silicone elastomer samples were heated to 115 °C, maintained at 115 °C for 5 min, and then heated to 300 °C at 10 °C/min. The blank uncured LSR-4350 silicone elastomer sample lost 0.3 % weight from 20 to 115 °C (solid line Fig. 3D). By comparison, the 10 % w/w milled DL-lactide sample lost 2.5 % weight at 115 °C (dashed line Fig. 3D), attributed to evaporation of a fraction (~22 %) of DL-lactide under the nitrogen atmosphere (50 mL/min); when the sample was heated further to 300 °C, practically all the initial lactide loading was lost. During manufacture, silicone elastomer rings are cured in heated and sealed ring mold tools. We do not know to what extent DL-lactide evaporates during ring manufacture. Content assay analysis for DL-lactide-loaded rings helped inform the extent of DL-lactide evaporation (Section 3.6.).

### 3.3. Differential scanning calorimetry analysis

DSC thermograms of milled DL-lactide powder samples and DL-lactide in cured silicone elastomer are presented in Fig. 3E. A melting peak with onset temperature of 124.8 °C was observed for the DL-lactide powder following the standard heat ramp protocol (Table 2). For the custom heating protocol (see Section 2.5; mimicking the thermal profile during ring manufacture), no thermal transitions were observed in the DSC thermogram during the first three stages, and subsequent melting of DL-lactide was observed at 124.7 °C during the final heat ramp stage (which involved heating above the crystalline melting temperature of DL-lactide), indicating that the crystalline structure of milled DL-lactide was unchanged after 5 min heating at 115 °C. A similar melting peak was observed at 124.4 °C and 124.5 °C in the thermograms of LSR-4350 silicone elastomer samples containing 20 % w/w DL-lactide (SE 20 % DL-lactide) and 30 % w/w DL-lactide (SE 30 % DL-lactide) (Table 2), indicating that (i) DL-lactide was chemically compatible with the silicone elastomer, (ii) curing the rings at 115 °C was not detrimental to the DL-lactide, and (iii) DL-lactide was present—at least partly (since some will inevitably be dissolved in the silicone matrix, otherwise release could not occur)—in the crystalline form within the rings, similar to other marketed silicone elastomer ring products.

### 3.4. Oscillatory rheology

**Fig. 4.**
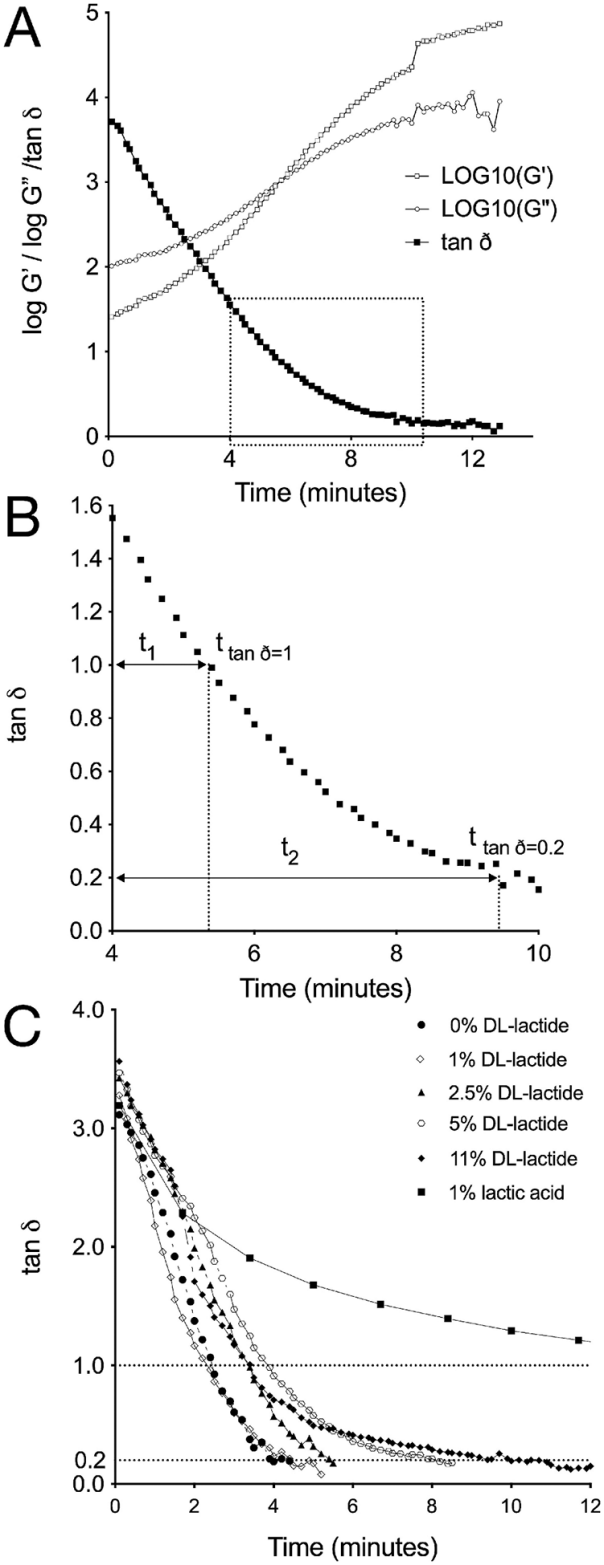
Representative oscillatory rheogram for (A) the isothermal cure at 60 °C of DDU-4320 silicone elastomer showing characteristic trends in storage modulus (G′), loss modulus (G″), and tanδ (=G″/G′) as a function of time. (B) Expanded region of (A) showing tanδ vs. time with *t*_tan *δ*=1_ and *t*_tan *δ*=0.2_ defined. (C) Tanδ versus time oscillatory rheograms showing the influence of DL-lactide and lactic acid on the cure characteristics (first 12 min only) of silicone elastomer DDU-4830 at 80 °C. Note that the sample containing 1 % w/w lactic acid significantly inhibits the elastomer curing reaction compared to that containing 1 % w/w DL-lactide.

Silicone elastomer curing reactions were monitored using oscillatory rheology (McConville et al., 2012, 2010). DDU-4320 was selected as a representative addition-cure silicone elastomer to assess the behaviours of DL-lactide and lactic acid. A representative rheogram showing the isothermal cure of DDU-4320 at 60 °C with storage modulus (G′), loss modulus (G″) and tan delta (tanδ) plotted as a function of cure time is presented (Fig. 4A). Increases in both G′ (a measure of the elastic nature of the material) and G″ (a measure of the energy dissipated as heat) were observed with time. Tanδ values—equal to G″/G′, a measure of the elasticity (tanδ < 1) or plasticity (tanδ > 1) of the sample—decreased with time due to formation of a crosslinked elastomer network. At the gel point, the values of G′ and G″ are equal and tanδ is 1 (Fig. 4A); the gel point represents the transition from a predominantly viscous liquid to a predominantly elastic material (McConville et al., 2010). Fig. 4B illustrates how values for time-to-tan *δ* = 1 (*t*_tan *δ*=1_) and time-to-tan *δ* = 0.2 *(t*_tanδ=0.2_), representing gel points and post-gel points respectively, were determined. For all formulations, tanδ values decreased with time due to the formation of crosslinked silicone elastomeric networks. Incorporation of DL-lactide (up to a loading of 11 % w/w) did not appear to significantly impact the silicone elastomer curing reaction (tan *δ* = 1 ranging from 2.3–3.3 min; times to tanδ = 0.2 ranging from 3.7–10.3 min) (Fig. 4C; Table 3). These data suggest that at a curing temperature of 80 °C, DL-lactide is compatible with the DDU-4320 silicone elastomer. By measuring these two parameters, the effect of DL-lactide and lactic acid incorporation and loading on the cure characteristics of the silicone elastomer at different cure temperatures was determined (Table 3). For the blank elastomer, time-to-tan *δ* = 1 ranged from 2.3–27.7 min and time to tan *δ* = 0.2 from 3.7–51.3 min. The lower cure temperatures (40 and 60 °C) are not practical for this elastomer since there is a preference for cure times ≤ 2 min. At 40 °C, formulations containing > 1 % w/w DL-lactide were unable to achieve full cure (tanδ = 0.2 was not achieved within the 80 min experiment). At cure temperatures of 60 °C and 80 °C, formulations containing DL-lactide showed increased time to tanδ = 1 and time to tanδ = 0.2 with increasing drug loading. Cure of samples containing 1 % w/w lactic acid at 60 °C and 80 °C was not achieved within 80 min. We conclude that lactic acid inhibits the elastomer curing reaction, and that it is not possible to practically manufacture silicone elastomer rings containing lactic acid. This has subsequently been confirmed using other addition-cure silicone elastomers, including LSR-4350. Thus, incorporation of DL-lactide as a lactic acid prodrug is a practical solution to overcoming this inhibition of crosslinking associated with lactic acid. We have previously reported inhibition of cure with other drug actives, most notably levonorgestrel whose alkynyl group competes with the hydrosilane groups that otherwise take part in the curing reaction (Dallal Bashi et al., 2019).

### 3.5. In vitro release testing

**Fig. 5.**
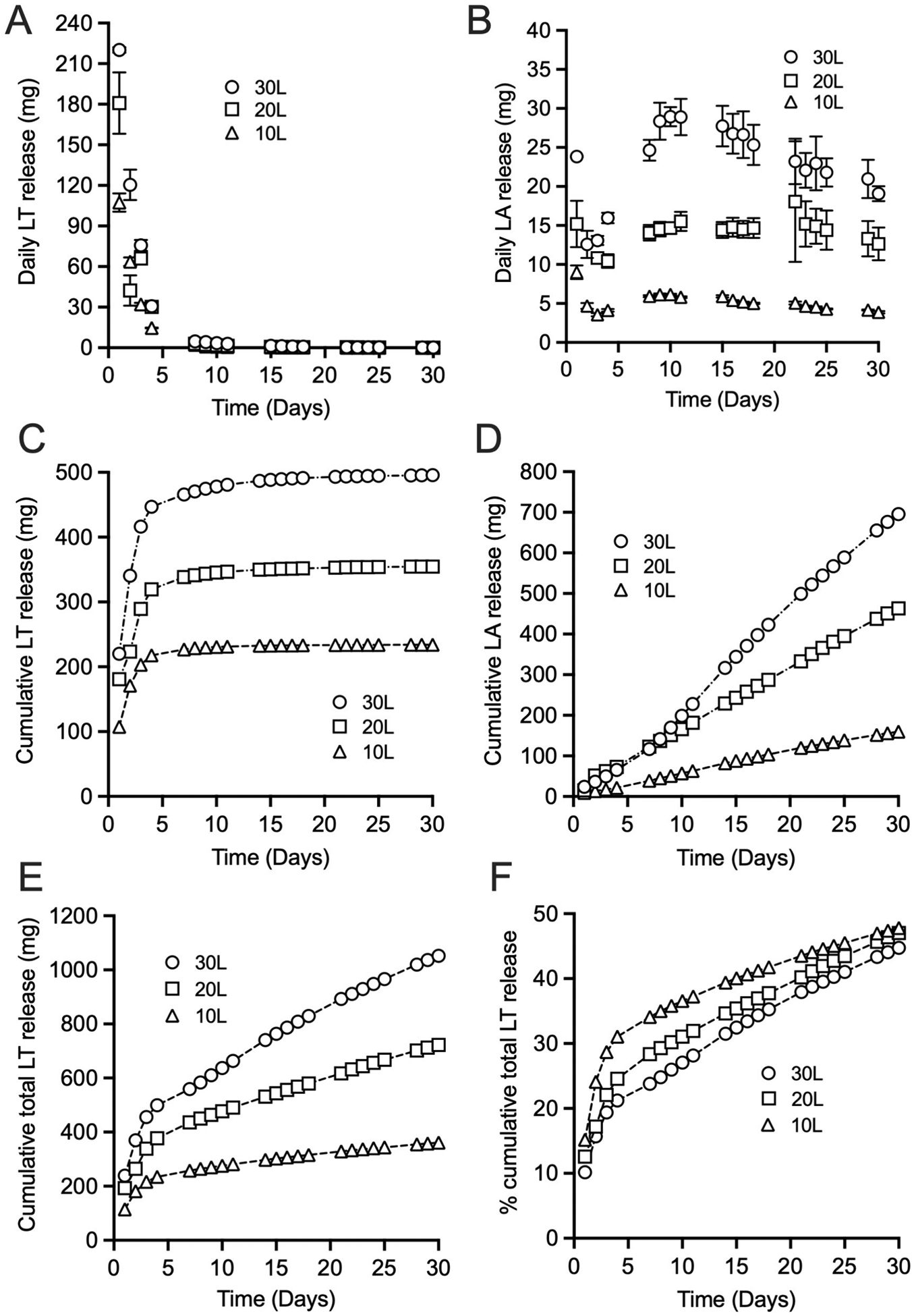
Mean daily lactide release (A), mean daily lactic acid release (B), cumulative lactide release (C), cumulative lactic acid release (D), cumulative total lactide release (E; values calculated by stoichiometric conversion of measured lactic acid release values to lactide release values and then adding the measured lactide release values) and percentage of cumulative total lactide release (F; based on panel E data) from DL-lactide loaded silicone elastomer LSR-4350 rings – 10L, 20L and 30L in deionized water over 30 days at 37 °C and 60 rpm. Each data point is the mean of three replicates and the error bars indicate the standard deviation in the daily release profiles; some error bars are smaller than the plot symbols.

DL-lactide was successfully released from the various LSR-4350 rings and detected in the deionized water release medium (Fig. 5A and 5C). The rings showed an initial burst release of lactide—ranging from 107 and 220 mg on Day 1 and correlating with initial lactide loading—followed by decreased lactide release on each subsequent day (e.g., Day 4 values ranged between 14.7 and 30.7 mg/day), and limited release from Day 7 onwards (Fig. 5A). The daily lactide release values during weeks 2–4 ranged from 4.73 to 0.05 mg/day. However, HPLC analysis of the release samples also revealed significant quantities of lactic acid out to Day 30 due to hydrolysis of the lactide (Fig. 5B and 5D). Two possible scenarios account for the presence of lactic acid in the release medium: (i) water is absorbed into the ring body, lactide is hydrolysed to lactic acid within the ring body, and the lactic acid is subsequently released from the ring via the diffusion; (ii) lactide is released from the ring via diffusion, and the lactide then hydrolyses to lactic acid in the release medium. It is difficult to determine the relative contribution of these two processes during the first week of *in vitro* release testing, since both lactide and lactic acid were measured in the release medium; however, after ~ 5 days *in vitro* release testing, the notable absence of DL-lactide in the release medium coupled with the continued release/detection of lactic acid out to Day 30 strongly suggests that lactic acid has become the dominant molecular species within the ring device.

It is worth commenting on the mechanistic aspects of hydrolysis of lactides in the human vagina. In general, esters—including lactones, such as lactides—undergo hydrolysis via three reaction mechanisms: (i) base-catalysed hydrolysis, (ii) acid-catalysed hydrolysis, and (iii) enzyme-mediated hydrolysis. Given the normal pH range of the healthy human vagina (3.8–4.5) (Owen and Katz, 1999) we expect acid-catalysed and enzyme-mediated hydrolysis of exogenously administered lactide to dominate. Various non-specific esterases are also present in the human vagina, including carboxylesterases, esterases derived from *Lactobacillus* species (and indeed other bacterial species), and hormone-dependent esterases. These play crucial roles in vaginal health, homeostasis, dysbiosis, and metabolism of drugs.

The quantities of lactic acid measured on Day 1 ranged 9–24 mg; release quantities then declined on Day 2 for all ring formulations, before increasing once again and peaking around Day 10 (6–29 mg, depending on initial lactide loading in the rings) (Fig. 5B). The daily lactic acid release values during weeks 2–4 ranged from 28.9 to 4.3 mg/day. The mean pH values of the release media over the 30-day *in vitro* release period were 2.6 ± 0.1, 2.7 ± 0.1 and 3.1 ± 0.1 for the L30, L20, and L10 rings, respectively, correlating with the initial lactide loadings in the rings and the *in vitro* release data.

Based on the stoichiometry of the lactide hydrolysis reaction, we converted the lactic acid release quantities into lactide and then plotted the total calculated amounts of lactide released from the rings (Fig. 5E). As anticipated, the total amount of lactide released correlated strongly with initial lactide loading. After 30 days, the percentage cumulative release values for lactide from L30, L20, and L10 rings were 44.7, 47.0 and 47.8 %, respectively (Fig. 5F). For comparison, residual levels of etonogestrel and ethinyl estradiol following 21 day clinical use of the contraceptive vaginal ring NuvaRing^®^ were 69–81 % (11.7 mg initial loading) and 79–93 % (2.7 mg initial loading), respectively (Haaland et al., 2017), and for the silicone elastomer contraceptive vaginal ring Progering^®^, ~40 % of the initial 2058 mg progesterone was released after up to 3 months continuous use (Murphy et al., 2019).

### 3.6. Lactide content assay

For the 30 % w/w lactide-loaded LSR-4350 rings, the mean lactide assay value post-manufacture—as measured by extraction and HPLC analysis, and after accounting for hydrolysis of lactide in the extraction medium—was 99.7 ± 0.6 %. We conclude that there was no significant change in lactide loading (w/w) during and following ring manufacture. This is supported by the TGA data (Section 3.2).

### 3.7. Ring weight and geometry

**Fig. 6.**
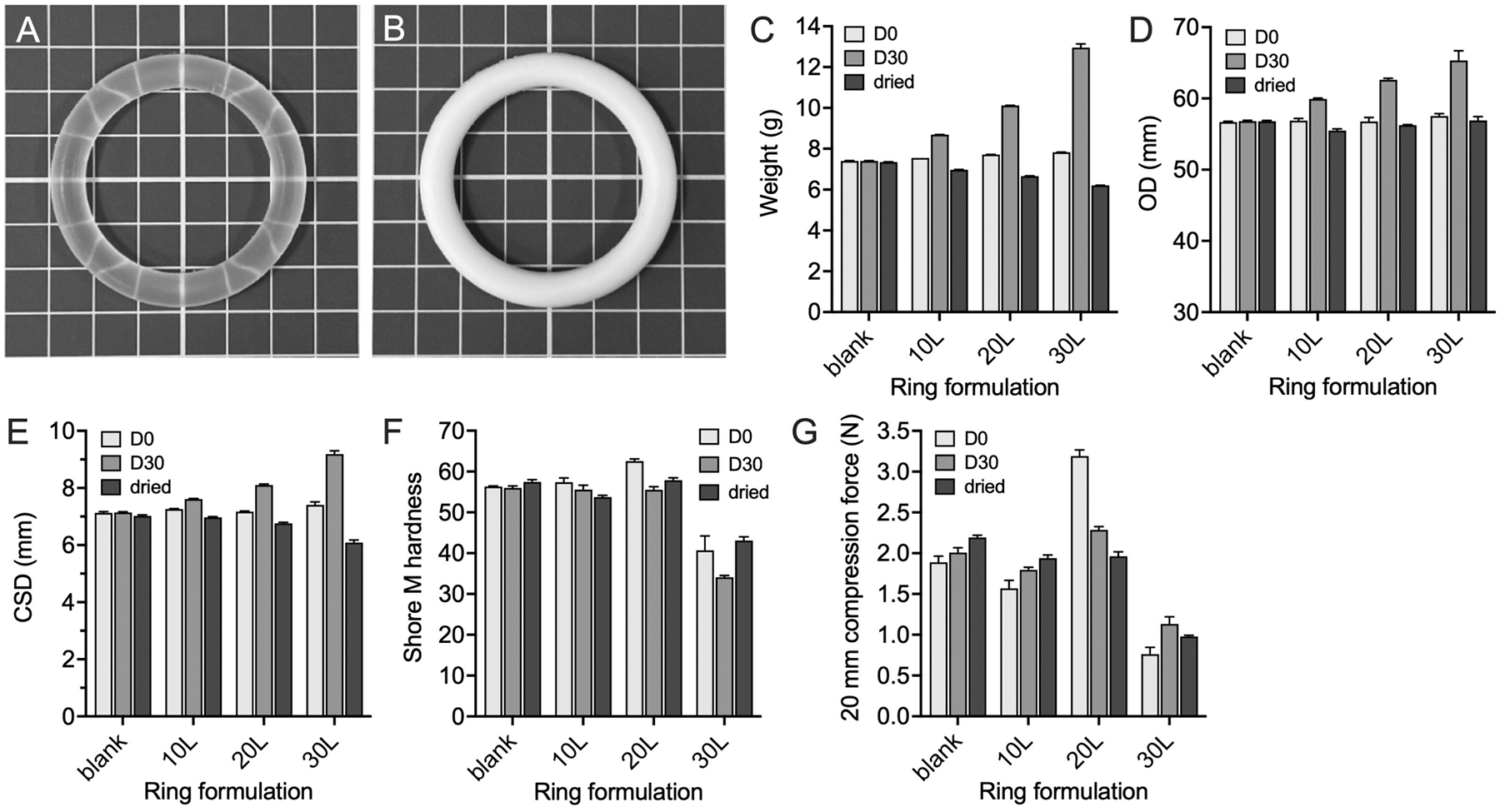
Representative images of matrix-type LSR4350 silicone elastomer vaginal rings: (A) blank ring, (B) ring containing 30 % w/w milled DL-lactide (30L). The squares in the background grid are 1 × 1 cm. Weights (C), outer diameters (OD) (D) and cross-sectional diameters (CSD) (E) of vaginal rings – blank, 10L, 20L and 30L before (D0) and after (D30) *in vitro* release testing and following subsequent drying at 50 °C for 21 days (dried) (n = 3). Error bars (included, but not always visible) represent the standard deviation of three replicates. Shore M hardness (F) and compression resistance at 20 mm compressive strain (G) for vaginal rings – blank, 10L, 20L and 30L before IVRT (D0), after 30-day IVRT in deionised water (D30) and after 21 day drying at 50 °C following IVRT (dried) (n = 3). Error bars indicate the standard deviation of three replicates.

Weights, outer diameters (OD) and cross-sectional diameters (CSD) of matrix-type LSR4350 silicone elastomer vaginal rings loaded with 0, 10, 20 and 30 % w/w lactide—before and after 30-day *in vitro* release testing, and after further drying for 21 days at 50 °C—are presented in Fig. 6. Initial ring weights increased from 7.40 g (blank) to 7.83 g (30 % w/w lactide) (Fig. 6C), ODs werê 57 mm (Fig. 6D), and CSDs ranged from 7.13–7.41 mm (Fig. 6E).

For blank rings, there was no significant difference in ring weight comparing D0 and D30 (p = 0.94). A 0.06 g loss in blank ring weight was observed comparing D0 and dried values (7.40 vs. 7.34 g), attributed to the loss of certain non-reactive volatile components in the silicone elastomer (Brook et al., 2007; Malcolm et al., 2016). Blank ring OD and CSD showed no differences between D0 and D30 (p = 0.34, p = 0.79); however, a slight decrease in CSD (from 7.13 mm D0 to 7.02 mm dried) was observed due to shrinkage during the 50 °C drying treatment (Bont et al., 2021). This drying was performed to force evaporation of water (*in vitro* release medium) from the silicone elastomer rings to better assess the influence of lactide/lactic acid release on the geometry and mechanical properties of the rings. Both loss of volatile components and shrinkage are common during post-cure of silicone elastomers (Andriot et al., 2007).

For the lactide-loaded rings, measured weights, ODs and CSDs were increased at D30 compared with D0 due to absorption of significant quantities of the water release medium (Fig. 6C–E), and the extent of increase correlated with the initial lactide loading. After drying to remove absorbed water, rings weights and CSDs decreased below the initial D0 values; therefore, these dried ring weights were indicative of the total release of lactide/lactic acid from the rings. The rank order of weight loss and the CSD decrease across the ring formulations—L10 < L20 < L30—correlated with the rank order of cumulative lactide release.

### 3.8. Durometer hardness

Durometer hardness is the international standard for measuring the hardness of rubber and plastic materials and has been used as a simple quality control test for matrix-type vaginal ring devices (Dallal Bashi et al., 2021; McCoy et al., 2019). The shore M hardness values for the various matrix-type vaginal ring formulations after manufacture (D0), after 30-day *in vitro* release testing in deionised water (D30), and after subsequent drying for 21 days at 50 °C (dried) are presented in Fig. 6F. Values ranged from 34 to 62, with values for D0 rings increasing significantly as initial lactide loading was increased from 0 to 20 % w/w, consistent with a filler effect (Mazurek et al., 2019). However, shore M hardness values for the L30 ring was significantly lower at all time-points, which we attribute to partial inhibition of the silicone elastomer curing reaction due to the presence of lactic acid. Under our laboratory conditions, exposure to atmospheric moisture and hydrolysis of lactide cannot be avoided during milling of the lactide powder and its subsequent mixing into the silicone elastomer parts, particularly at this highest 30 % w/w lactide loading. The hydrosilylation reaction that occurs with addition cure silicone elastomers involves reaction between hydride- and vinyl-functionalized polydimethylsiloxanes (Malcolm et al., 2016). However, the carboxylic acid of the lactic acid molecule and the silane (Si–H) groups in the silicone elastomer undergo dehydrogenative coupling to form silyl esters in the presence of the platinum catalyst (Köllnberger et al., 2020), thereby competing with the normal crosslinking reaction and leading to reduced crosslinking density.

There was no significant difference in hardness values for blank rings at D0 and D30 (p = 0.56), although increased hardness values were recording after drying (p = 0.03) due to extended cure caused by the high temperature condition. Swelling of the lactide-loaded rings at D30 resulted in decreased hardness compared with D0. L10 and L20 rings showed decreased hardness after drying, while L30 rings showed increased hardness after drying. The net hardness changes measured with dried lactide rings were due to lactide depletion from the rings and post-curing effects.

### 3.9. Ring compression test

The compression test is one of the most important *in vitro* mechanical tests for vaginal ring products (Boyd et al., 2020a; McCoy et al., 2019). Although different compression distances may be used, we now routinely use 20 mm. Compression force data for matrix-type LSR4350 rings loaded with 0–30 % w/w lactide—after manufacture (D0), after *in vitro* release testing (D30) and after drying (dried)—are presented in Fig. 6G. For D0 rings, the rank order for compression force was: L20 (3.19 N) > blank (1.89 N) > L10 (1.57 N) > L30 (0.76 N). For blank and L10 rings, the force increased (D0 < D30 < dried) due to post cure effects (Lee et al., 2016). However, the reverse trend was observed for the L20 ring—D0 (3.19 N) > D30 (2.29 N) > dried (1.96 N), due to an increased mechanical filler effect associated with the higher lactide loading and the increased quantity of lactide released from the ring. The lowest compression forces were measured for the L30 ring, correlating with hardness values (Fig. 6F) and due to lactic acid cure inhibition.

Compression forces have previously been reported for certain marketed drug-releasing vaginal rings (Femring^®^, NuvaRing^®^ and Eatring^®^) (McCoy et al., 2019), and the importance of compression to user comfort and involuntary expulsion has been discussed (Boyd et al., 2020b). The extent to which ring flexibility (compressibility) impacts user acceptability, comfort and adherence is still unknown, although ongoing clinical studies will provide insights.

### 3.10. Anti-sperm activity of lactide and lactic acid

Incorporation of lactide as a lactic acid prodrug could be useful in developing new MPTs. Because sperm might only be exposed to compounds released from a vaginal ring for a short time period before they enter and transit through the cervix, we tested the effects of different concentrations of DL–lactide and L–lactic acid on sperm motility at the 5 min timepoint. For these studies, the percent motility refers to all types and quality of sperm movement, ranging from vibrating/twitching with no forward progression at all, to moving progressively. In contrast, progressive motility refers to sperm that are actively moving in a straight line or in large purposeful circles. Progressive motility is required for normal sperm function including penetration of cervical mucus.

**Fig. 7.**
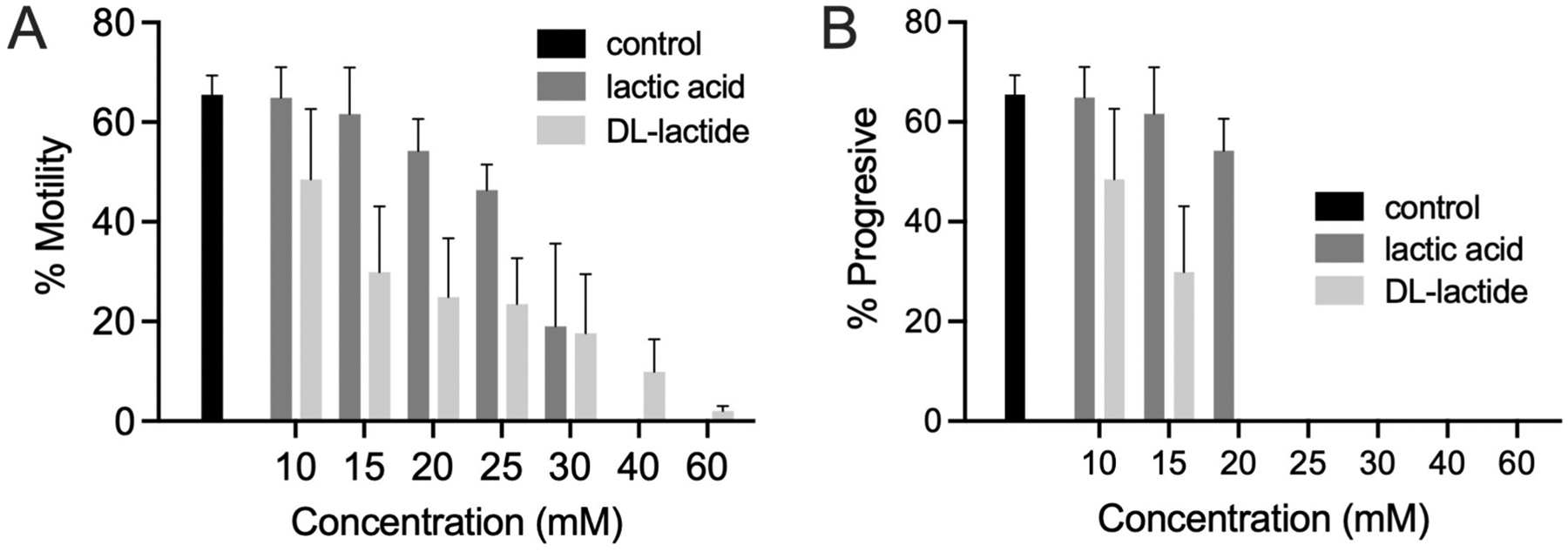
Anti-sperm activities of lactide and lactic acid, as measured by % motility (A) and % progression (B).

The saline solutions of lactic acid and DL-lactide tested in the sperm motility assay were acidic, with pH values dependent upon their concentrations. DL-lactide was slightly more effective at most concentrations tested, but not by a significant margin (Fig. 7). The calculated EC_50_ values for total motility were 19.8 mM for DL-lactide and 24.9 mM for lactic acid. Based on non-linear regression, the EC_90_ values are estimated to be 42.4 mM for lactic acid and 37.7 mM for DL-lactide. Overall, DL-lactide does appear to be slightly more effective than lactic acid. This is encouraging, as activity is not solely dependent upon lactide hydrolysis to lactic acid. Given that lactide fully hydrolyses in solution to form two lactic acid molecules, it was expected that it would provide twice the anti-sperm activity of lactic acid at equimolar concentrations. That this was not observed may be due to lactide only being partially hydrolysed within the timeframe of these anti-sperm tests; for example, lactoyllactic acid forms as an intermediate product in the hydrolysis of lactide (Diaz et al., 1995).

**Fig. 8.**
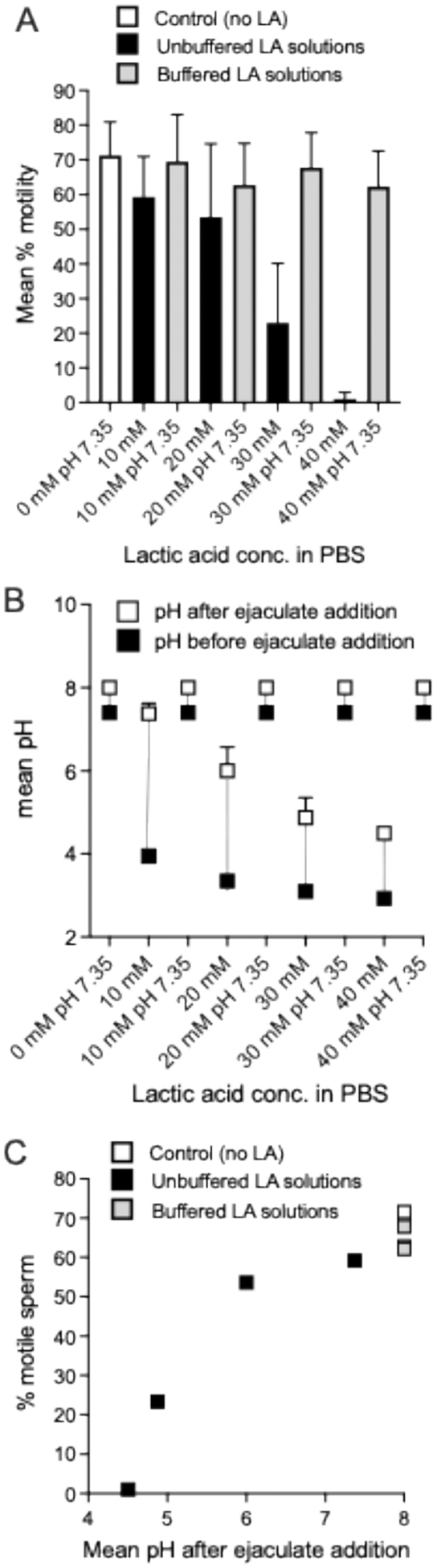
The effects of different lactic acid concentrations on sperm motility and pH of seminal plasma samples under pH-controlled (7.35) and pH-uncontrolled conditions. (A) Mean % motility after 5 min incubation with control or lactic acid solution (n = 4 separate donors), error bars represent SEM. (B) Change in pH before and after addition of aliquot of semen to lactic acid or control solution. (C) Association between mean final pH of the sample and % motile sperm after 5 min incubation with lactic acid or control (n = 4).

It is worth commenting briefly on the potential mechanism by which lactide/lactic acid inhibit sperm motility. Lactate (the ionized form of lactic acid) is an excellent energy source for sperm, and human sperm have membrane proteins that transport monocarboxylates, such as lactate/lactic acid (Amaral, 2022; Brauchi et al., 2005). We suspect that the non-ionized weakly acidic species (in their protonated form) formed during DL-lactide hydrolysis—primarily lactic acid, but potentially also the intermediate lactoyllactic acid—are inhibiting sperm motility by significantly reducing pH and reducing Na+/K + -ATPase activity (Zhou et al., 2015). In support of this, we found that when the lactic acid solution pH was maintained at 7.35, we observed significantly less motility inhibition (Fig. 8A). Even at a concentration of 40 mM lactic acid, sperm maintained an average motility similar to controls when the pH was held constant at 7.35, compared to almost complete motility inhibition under uncontrolled pH conditions (Fig. 8A). Additionally, we found that the dose-dependent effect of lactic acid on sperm motility was associated with concurrent dose-dependent decrease in pH of the aliquot of semen to which the lactic acid was added (Fig. 8B–C). Higher concentrations of lactic acid were better able to overcome the buffering capacity of the seminal plasma, causing a greater decrease in pH and greater resulting decline in sperm motility (Fig. 8). These results support that the observed effects of lactic acid on sperm motility were primarily pH dependent.

### 3.11. Virucidal activity of lactic acid against HSV-2 and HIV-1BaL

**Fig. 9.**
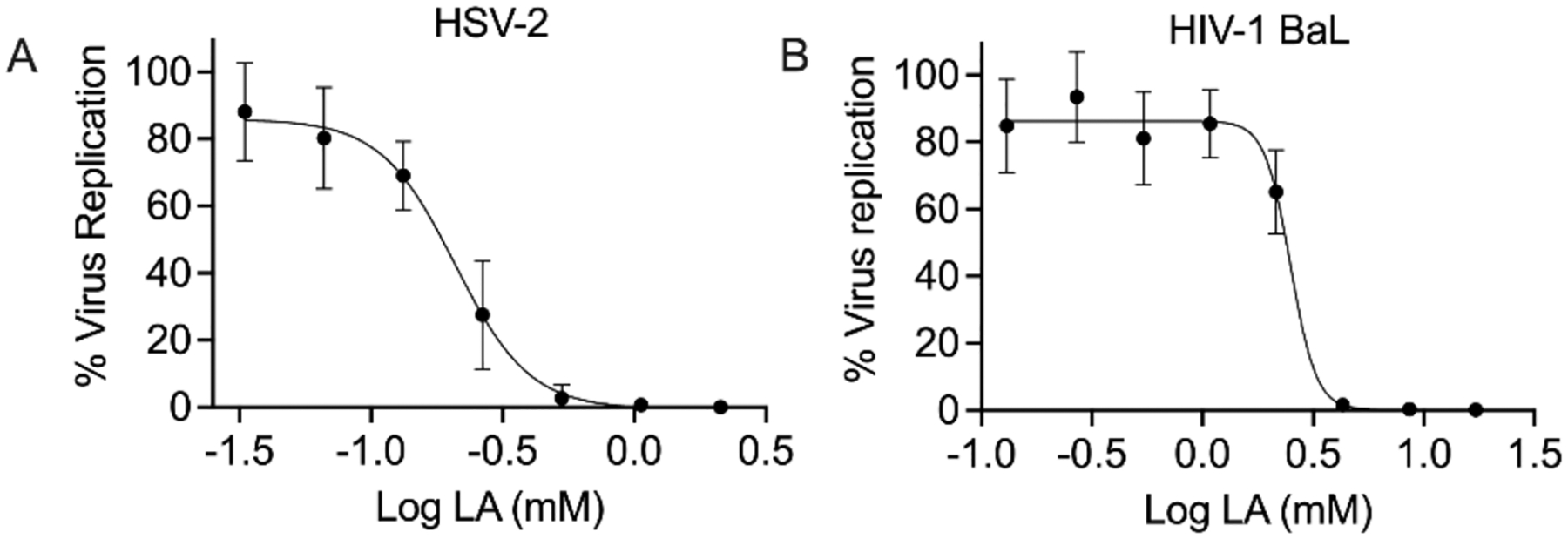
Virucidal activity of lactic acid (LA) against HSV-2 and HIV-1BaL. HSV-2 and HIV-1BaL were pre-incubated with different LA concentrations and titered in Vero cells and TZMbl cells, respectively. The graph shows the percent of virus replication versus the log concentration of acid lactic. (A) HSV-2: Each data point represents the average of 6 replicates from two independent experiments. (B) HIV-1BaL: Each data point represents the average of 9 replicates from three independent experiments.

The virucidal activity of lactic acid against HSV-2 and HIV-1BaL is presented in Fig. 9. Lactic acid inhibits HSV-2 replication in a dose-dependent manner and shows an EC_50_ of 0.2 mM with a 95 % confidence interval between 0.17 and 0.24 mM. Our results confirm previously reported data (Conti et al., 2009) with EC_50_ values in the low mM range for lactic acid. Similarly, lactic acid inhibits HIV-1BaL replication in a dose-dependent manner with an EC_50_ of 2.5 mM. Prism was unable to calculate a complete 95 % confidence interval. We observed complete or nearly complete inhibition of HIV-1BaL infection following exposure to 4.3–17.2 mM lactic acid. These results are consistent with previous reports (Aldunate et al., 2015). These observed antiviral activities of lactic acid are likely due to (i) pH lowering which damages the virus and blocks attachment/entry (e.g., disruption of the viral envelope, denaturing of envelope glycoproteins, and interference with the conformational changes needed for membrane fusion), and (ii) a direct virucidal effect whereby the undissociated form of lactic acid penetrates the viral envelope and disrupts internal structures.

### 3.12. Anti-bacterial activity of lactic acid

**Fig. 10.**
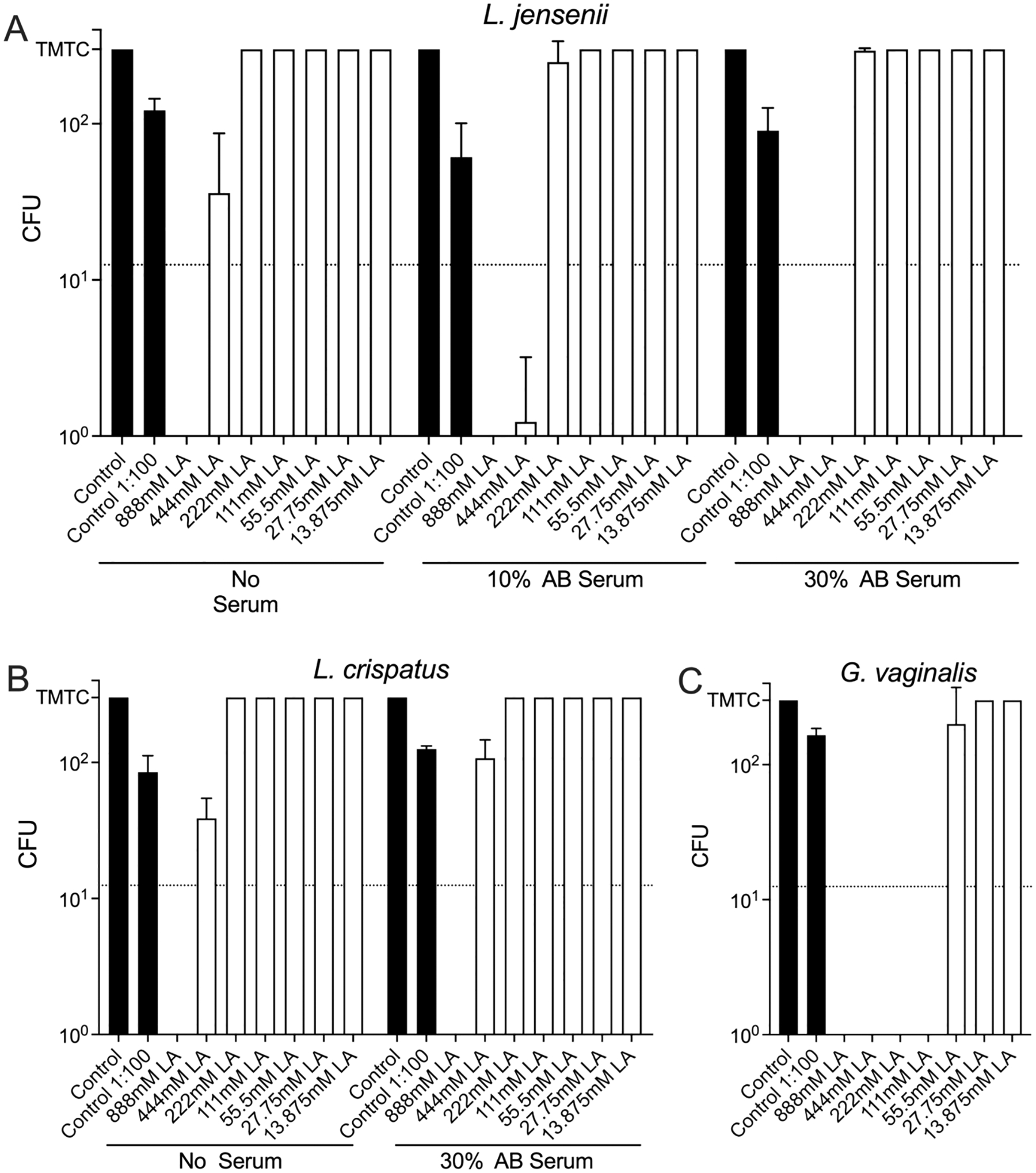
Anti-bacterial activities of lactic acid (LA). Bacteria were pre-incubated with different concentrations of LA ± AB serum and then plated on agar plates and cultured anaerobically. Plates with > 300 CFU were considered too many to count (TMTC) and set as 300 colonies. The dotted lines (12.5 colonies) represent 99.9 % inhibition. Shown are CFU summaries (mean ± SD) from two or three experiments (A) and three experiments (B, C).

Lactic acid at physiological concentrations (111 mM) (Aldunate et al., 2013; O’Hanlon et al., 2011) has bactericidal activity against *G. vaginalis*, but not against *Lactobacillus* (Fig. 10). At high supra-physiological concentrations, lactic acid showed bactericidal activity against *L. jensenii* and *L. crispatus*. The activity in the presence or absence of AB serum in anti-*Lactobacillus* assay was similar. Lactic acid’s selective antibacterial effect against *G. vaginalis* but not Lactobacillus is likely due to a combination of pH tolerance, membrane physiology, and metabolism. Unlike lactobacillus, *G. vaginalis* is poorly adapted to survive acidic environments; it thrives best in near neutral pH environments (such as those observed with bacterial vaginosis) and is inhibited at acidic pH values (typical of a healthy human vagina). Also, lactic acid in its undissociated form can penetrate *G. vaginalis* cell membranes, acidifying the cytoplasm and disrupting essential enzyme systems. Finally, unlike *G. vaginalis,* most *Lactobacillus* species produce lactic acid as a major metabolic end product of carbohydrate fermentation.

## 4. Conclusions

The ability to incorporate DL-lactide into an addition-cure silicone elastomer vaginal ring as a means of providing sustained release of lactic acid is an important achievement, particularly given (i) the incompatibility of lactic acid with such elastomers, (ii) the physiological importance of lactic acid in the healthy human vagina, (iii) the relatively low release rates for lactic acid reported previously for thermoplastic rings, and (iv) growing interest in developing simple, inexpensive and practical MPT vaginal rings having contraceptive, antiviral and antibacterial activities. The work described here lays the foundation for development of new silicone elastomer ring formulations—and particularly MPTs—for which release of lactic acid might usefully be combined with release of other actives.

## Figures and Tables

**Table 1:** Marketed vaginal products containing lactic acid.

Product (Manufacturer)	Dosage Form	Administration Dose (LA Content)	Frequency of Dosing	Use
GynoFit^®^ Lactic Acid Gel (Tentan AG Switzerland)	Gel	5 mL*	Apply as necessary	Regulates vaginal pH / Vaginal infection & BV symptom relief
Relactagel (Kora Healthcare)	Gel	5 mL (4.5 % / 225 mg)	Acute: Once daily for 7 days Prevention: 1 tube for 2–3 days after menstrual period	BV treatment / prevention
Phexxi^™^ (Evofem Biosciences Inc.)	Gel	5 g (1.8 % / 90 mg)	1 applicator up to 1 h prior to intercourse	On-demand contraceptive (Thomas et al., 2020)
Vagisan Lactic Acid (Dr. August Wolff GmbH & Co.)	Pessary	Single pessary (40 mg per pessary)	1 pessary daily for 5–7 consecutive days or 1 pessary 2–3 times a week for longer term use	Regulates vaginal pH / prevention vaginal infections (Plummer et al., 2021b)
Balance Activ^®^ BV Treatment Gel (Venture Life Group plc)	Gel	5 mL*	1 tube daily for 7 days	Maintain healthy vaginal pH / BV treatment
Balance Activ^®^ BV Treatment Pessary (Venture Life Group plc)	Pessary	Single pessary*	1 pessary daily for 7 days	Maintain healthy vaginal pH / Treatment of BV
Canesbalance^®^ BV Gel (Bayer plc)	Gel	5 mL*	1 tube daily for 7 days	Vaginal infections / BV
Canesbalance^®^ Vaginal Pessaries (Bayer plc)	Pessary	Single pessary*	1 pessary daily for 7 days	Vaginal infections / BV treatment
Restore^®^ Moisturizing Vaginal Gel (Good Clean Love Inc)	Gel	4–5 g*	1 application for 3 consecutive days followed by 1–2 applications per week	Vaginal moisturizer / Regulates vaginal pH

* Lactic acid concentration not stated by manufacturer.

**Table 2:** Thermal parameters for each melting transition in DSC thermograms for milled DL-lactide powder samples and LSR-4350 silicone elastomer samples loaded with milled DL-lactide (n = 3, mean ± sd).

	Onset Temp. (°C)	Enthalpy (ΔH, Δ/g)
DL-lactide (ramp heating)	124.83 ± 0.04	151.77 ± 2.18
DL-lactide (custom method)	124.74 ± 0.01	165.68 ± 7.16
SE 10 % w/w DL-lactide	124.74 ± 0.09	13.19 ± 0.77
SE 20 % w/w DL-lactide	124.35 ± 0.09	29.03 ± 0.48
SE 30 % w/w Dl-lactide	124.46 ± 0.21	45.42 ± 0.50

**Table 3:** DDU-4320 silicone elastomer formulations and their accompanying *t*_tan *δ*=1_ and *t*_tan_ *δ*=0.2 values. Experiments were conducted for 80 min, *t*_tan_ *δ*=1 values are shown in bold, *t*_tan *δ*=0.2_ in italics, ‘–’ represents replicates that did not achieve tanδ values within this time frame.

Formulation	Time (min) to tan_δ = 1.0, *0.2*_^a^		
	Cure Temperature (°C)		
	40	60	80
Blank	**27.7 ± 2.1**	**5.3 ± 0.1**	**2.3 ± 0.1**
	*51.3* ± *0.8*	*9.8* ± *0.1*	*3.7* ± *0.7*
1 % DL-lactide	**51.1 ± 0.1**	**11.8 ± 1.9**	**2.3 ± 0.9**
	–	*34.0* ± *0.5*	*4.2* ± *1.1*
2.5 % DL-lactide	–	**14.2 ± 0.4**	**2.9 ± 1.0**
	–	*42.7* ± *0.5*	*4.8* ± *0.8*
5 % DL-lactide	–	**18.2 ± 2.8**	**3.5 ± 0.9**
	–	*55.5* ± *0.6*	*8.5* ± 1.2
11 % DL-lactide	–	**32.5 ± 0.8**	**3.3 ± 0.7**
	–	–	10.3 ± *0.6*
1 % lactic acid	–	–	**18.1 ± 1.7**
	–	–	–

a Determined from tanδ vs time plots, according to Fig. 4B.

## Data Availability

Data will be made available on request.
